# The role of telehealth models, including teleneuropsychology, in assessing decision-making capacity in older adults with cognitive impairments: a scoping review

**DOI:** 10.3389/fpsyg.2026.1798682

**Published:** 2026-04-21

**Authors:** Maneesh Varghese Kuruvilla, Angela Blazely, Matthew So, Alisa Green

**Affiliations:** 1 School of Psychological Sciences, Macquarie University, Sydney, NSW, Australia; 2 Wicking Dementia Research and Education Centre, University of Tasmania, Hobart, TAS, Australia; 3 Tasmanian Health Service, Hobart, TAS, Australia; 4 Department of Aged Care and Rehabilitation, Liverpool Hospital, Sydney, NSW, Australia

**Keywords:** cognitive assessment, dementia, guardianship, telehealth, telemedicine

## Abstract

**Objective:**

Decision-making capacity evaluations are decision-specific and typically rely on integrated evidence from interview-based assessments of decisions, functional abilities, collateral history, and behavioural observations, with cognitive testing providing supportive (non-determinative) evidence. Telehealth models, including teleneuropsychology, have potential to expand access to capacity-related assessments for older adults with cognitive impairments who cannot attend in-person services, but its use for decision-making capacity assessments remains unclear. This scoping review mapped how telehealth has been used to support decision-making capacity assessments, explicitly or implicitly, in older adults with cognitive impairments and summarised feasibility, validity/reliability, and implementation considerations.

**Method:**

A scoping review was conducted in accordance with PRISMA-ScR guidance. Five databases were searched from inception to 05 January 2026. Studies were eligible if they involved older adults with cognitive impairments (aged ≥65 years) and used telehealth for assessment processes that either evaluated decision-making capacity (Tier A), or informed capacity-adjacent decisions (e.g. driving, living alone, guardianship planning; Tier B).

**Results:**

The search produced 1,738 results; a total of 1,264 records were screened, and 102 full-text articles were retrieved for further review. Five studies met inclusion criteria, three of which were classified as Tier A for at least one patient. The remaining two studies primarily involved telehealth cognitive, geriatric, or psychiatric assessments that informed capacity-adjacent recommendations but did not operationalise decision-making capacity as a primary outcome. All telehealth models were supported by additional staff, with onsite staff supporting technology and, in some cases, components of the assessments. Reported opportunities included improved access and timeliness; barriers included sensory impairment, variable infrastructure, and limited reporting in tele-specific adaptations and decision-specific capacity procedures.

**Conclusion:**

Direct evidence for telehealth-delivered decision-making capacity assessments in older adults with cognitive impairments remains limited. Studies of telehealth capacity assessments involving teleneuropsychology are even more limited. Future work should prioritise clear operationalisation and reporting of decision-specific decision-making capacity procedures, applied frameworks, guidelines and policies, as well as telehealth delivery models (including facilitation), and outcomes addressing feasibility, reliability/validity, and stakeholder acceptability. The necessity for telehealth neuropsychology-specific normative data also needs to be evaluated.

## 1 Introduction

Decision-making capacity refers to an individual’s ability to make a specific decision at a particular time (Appelbaum, 2007; Stewart and Rock, 2013). Four key consensus principles are typically applied when *clinically* assessing decision-making capacity across various clinical-legal frameworks, jurisdictions, settings and disciplines. These commonly take the form of determining a person’s ability to (1) understand relevant information, (2) appreciate how that information applies to their circumstances, (3) reason about options and consequences, and (4) communicate a stable choice (Australian Psychological Society, 2015; British Psychological Society, 2019; ABA/APA Assessment of Capacity in Older Adults Project Working Group, American Bar Association. Commission on Law, Aging and American Psychological Association, 2008; Grisso and Appelbaum, 1998; Wong et al., 2024; Mooney et al., 2024; Wood et al., 2020). Decision-making capacity is therefore risk-, decision- and time- specific, and it may fluctuate with delirium, psychiatric symptoms, medication effects, and changes in medical status. In older adults with cognitive impairments, decision-making capacity assessments often require careful attention to communication and functional barriers (e.g. aphasia, hearing/vision impairment), cultural and educational factors, and the availability and reliability of collateral information (Australian Psychological Society, 2015; British Psychological Society, 2019; ABA/APA Assessment of Capacity in Older Adults Project Working Group, American Bar Association. Commission on Law, Aging and American Psychological Association, 2008). It is consistently argued that emotion and personal values should also be considered component aspects of clinical assessments for decision-making capacity (Australian Psychological Society, 2015; British Psychological Society, 2019; Wood et al., 2020; Hermann et al., 2016).

Assessing capacity can be an intensive and exacting process, with significant personal and legal consequences. It involves managing a nuanced balance between maintaining an individual’s autonomy and protecting them from apparent or potential neglect, abuse, risk, and exploitation (Purser, 2017; Bennett, 2016). Clinicians are guided by various legal requirements, as well as discipline and professional standards, policy guidelines, and clinical frameworks when assessing an individual’s ability to make informed decisions (Wong et al., 2024; Wood et al., 2020; Chiu et al., 2017; Peisah et al., 2013). Neuropsychologists frequently contribute to capacity-related evaluations by detailing cognitive strengths and weaknesses (e.g. attention, memory, language, executive functioning) that may support or undermine decision-making abilities (Wood et al., 2020; Wood et al., 2007; Mullaly et al., 2007), and how this is impacting the decision-making process specific to the situation at hand. Cognitive impairment is one of the strongest predictors of impaired capacity (Gurrera et al., 2006; Okonkwo et al., 2008; Moye and Marson, 2007; Okonkwo et al., 2007). As such, largely because of an increased risk of developing cognitive impairments (e.g. dementia) and the impact of this on capacity, older adults can be particularly vulnerable to abuse and neglect. Importantly, though, cognitive test performance and/or neuropsychological functioning are not interpreted as a pass/fail threshold for capacity, nor should such be interpreted in isolation. Furthermore, there is no standalone test or evaluative tool that can determine someone’s capacity to make informed autonomous decisions. Capacity opinions are typically and by necessity based on an integrated synthesis of interview-based assessments of a specific decision, functional abilities, collateral history, and behavioural observations, with cognitive testing providing supportive evidence that helps explain why an individual may struggle with the decision demands (Australian Psychological Society, 2015; British Psychological Society, 2019; ABA/APA Assessment of Capacity in Older Adults Project Working Group, American Bar Association. Commission on Law, Aging and American Psychological Association, 2008; Grisso and Appelbaum, 1998; Wood et al., 2020); neuropsychologists typically receive training in the considered integration of all these areas over and above just completing cognitive testing (Wong et al., 2024; Mullaly et al., 2007). Professional competency codes and requirements regarding neuropsychology standards for capacity assessments are frequently recommended (Australian Psychological Society, 2015; British Psychological Society, 2019; Mullaly et al., 2007).

Translating capacity-related assessment processes to telehealth is potentially valuable, particularly for older adults living in rural areas, those with mobility limitations, and people who cannot attend in-person specialist services (Bilder et al., 2020). Beyond access, telehealth may improve timeliness and enable greater standardisation through scripted protocols and structured documentation (Rush et al., 2022). However, capacity assessments present telehealth-specific challenges, including consent for the assessment, managing sensory impairment and communication supports, ensuring privacy, and clarifying which components are delivered remotely versus those facilitated onsite by staff. These issues are especially relevant because much telehealth work with older adults occurs in facilitated or hybrid models (e.g. rural hospitals or nursing facilities) rather than direct-to-home, unassisted assessment (Qi Tan et al., 2022).

Accordingly, the aim of this scoping review was to map if and how teleneuropsychology and related telehealth assessment models have been used to support decision-making capacity assessments in older adults with cognitive impairments, and to summarise feasibility, reliability/validity, and implementation considerations. This review focused specifically on older adults with cognitive impairments because dementia and age-related cognitive decline are among the most common clinical contexts in which decision-making capacity concerns arise. Additionally, telehealth-supported capacity assessment in younger populations may involve substantially different referral questions, etiologies, and service models, including acquired brain injury, intellectual disability, primary psychiatric conditions, and forensic contexts, which were outside the scope of the present review. While there are reviews exploring how teleneuropsychology can facilitate the remote administration of cognitive assessments (Carlew et al., 2020; Walker et al., 2023; Brown and Zakzanis, 2025; Brearly et al., 2017; Grosch et al., 2011; Sperling et al., 2024), including in older adults with cognitive impairments (Friedman et al., 2025; Salvadori et al., 2023; Binng et al., 2021; Cullum et al., 2006; Wadsworth et al., 2018; Carotenuto et al., 2021), there is no published review examining how capacity assessments have been and can be conducted using telehealth. We distinguished between studies that explicitly assessed decision-making capacity for defined decisions/domains (Tier A) and studies in which telehealth assessments informed capacity-adjacent recommendations but did not operationalise decision-making capacity as the primary outcome (Tier B).

## 2 Methods

### 2.1 Identifying the research question

The Population-Concept-Context (PCC) framework (Peters et al., 2022) was used to identify the main concepts and search terms (Table 1) to help establish the following research questions:

- Is teleneuropsychology used to facilitate capacity assessments for older adults with cognitive impairments? If yes, is it an appropriate/valid method?
- Does teleneuropsychology have the potential to increase access to capacity assessments?
- What are the potential opportunities for and barriers to the adoption of teleneuropsychology capacity assessments?

**Table 1:** PCC framework for identifying key concepts of the scoping review.

PCC framework	Term	Definition
Population	Older adults with cognitive impairments	*“Those aged 65 years and over”* (Australian Institute of Health and Welfare (AIHW), 2023) with *“deficits in neurocognitive domains. Deficits can be defined as a difference from baseline, difference from age and education matched controls, or difference from the level of other neurocognitive domains in an individual”* (Richey and Peters, 2025)
Concept	Assessment of capacity	*“...the patient’s understanding of the relevant information, the patient’s appreciation of the significance of this information for the circumstances, the patient’s ability to reason with the relevant information and weigh options logically, and the patient’s ability to express a choice.”* (Hurst, 2004)
Context	Teleneuropsychology methods	*“The application of audiovisual technologies to enable remote clinical encounters with patients to conduct neuropsychological assessments.”* (Bilder et al., 2020)

### 2.2 Identifying the relevant studies

Relevant studies were shortlisted using the systematic search strategy described below and with the help of the PRISMA-ScR Checklist (Tricco et al., 2016) (Supplementary Table 1). This strategy was developed in consultation with a librarian at the University of Tasmania, Michaela Venn. Five databases – PubMed, PsycInfo, CINAHL, Web of Science and Google Scholar - were used to search for relevant literature. Table 2 showcases the template used to shape the selection of keywords in a PubMed database search. Similar templates were used for all selected databases. The concept of ‘older adults/aged’ was excluded from the search template to minimise the chance of missing studies that did not explicitly include these terms in the title/abstract and to account for the assumption that capacity assessments related to cognitive impairments are often conducted with older adults.

**Table 2:** Template used to identify key concepts and search terms in PubMed.

Concept no	Concept 1	Concept 2	Concept 3
Key concepts	Teleneuropsychology	Capacity assessment	Cognitive impairment
Free text terms/natural language terms	Healthcare access	Cognitive assessment	Cognitive impairment
	Telehealth	Decision-making capacity	Dementia
	Videoconference*	Capacity assessment	Alzheimer’s disease
		Neuropsychological assessment	Cognitive decline
	Telemedicine	Neuropsychological test*	
	Teleneuropsychology		
	Teleneuropsychology		
Controlled vocabulary terms/subject terms	Telemedicine; videoconferencing	Neuropsychological tests	Cognitive dysfunction; dementia

The asterisk (*) was used as a truncation symbol to capture word variants sharing the same stem in the search strategy.

Table 3 provides a summary of the search strategy for the PubMed database using the keywords identified in Table 2. Search strategies for the remaining four databases have been provided in Supplementary Tables 2–5. The database searches were initially conducted on 16 Apr 2024 and were updated on 05 Jan 2026 prior to submission to capture newly indexed records. The update used the same search strategy, restricted to records published/indexed from 2024 to 2026. For Google Scholar, only the first 200 records – sorted by relevance – were included in the full list of identified records; this is a number that has been identified to generate a 95% recall of relevant papers (Bramer et al., 2017).

**Table 3:** PubMed search history results.

Search number	Query	Results on 05/01/2026
1	(((((((Telemedicine[MeSH Terms]) OR (videoconferencing[MeSH Terms])) OR (tele-neuropsychology[Title/Abstract])) OR (teleneuropsychology[Title/Abstract])) OR (telemedicine[Title/Abstract])) OR (telehealth[Title/Abstract])) OR (videoconferenc*[Title/Abstract])) OR (healthcare access[Title/Abstract])	87,936
2	(((((Neuropsychological tests[MeSH Terms]) OR (neuropsychological test*[Title/Abstract])) OR (neuropsychological assessment[Title/Abstract])) OR (capacity assessment[Title/Abstract])) OR (decision-making capacity[Title/Abstract])) OR (cognitive assessment[Title/Abstract])	225,951
3	(((((Cognitive dysfunction[MeSH Terms]) OR (dementia[MeSH Terms])) OR (cognitive impairment[Title/Abstract])) OR (dementia[Title/Abstract])) OR (Alzheimer’s disease[Title/Abstract])) OR (cognitive decline[Title/Abstract])	444,206
4	#1 AND #2 AND #3	243

The asterisk (*) was used as a truncation symbol to capture word variants sharing the same stem in the search strategy.

# denotes the PubMed search history set number.

### 2.3 Study selection

All records were imported into Covidence for deduplication and screening (Babineau, 2014). Titles/abstracts were screened against eligibility criteria (Table 4) by MVK, followed by full-text review. Studies were eligible if they involved older adults with cognitive impairments (average cohort age ≥65 years) and used telehealth for assessment processes that could be classified as either Tier A or Tier B. A subset of records (5%) was independently screened by AB, MS, and AG to verify consistency. Any disagreements were resolved by discussion. If consensus could not be reached, a third reviewer adjudicated. There was 98.5% agreement between reviewer and verifiers for the title/abstract and 100% agreement for the full-articles. For the 05 Jan 2026 update, titles and abstracts of newly indexed records were screened against the same eligibility criteria. As no potentially eligible new studies were identified, full-text retrieval and duplicate data extraction were not undertaken for the updated search.

**Table 4:** Inclusion/exclusion criteria.

Inclusion criteria	Exclusion criteria
Individuals >65 years old	Studies that use teleneuropsychology primarily for providing assessment feedback or conducting rehabilitation interventions
All studies that specify the use of teleneuropsychology to assess decision-making capacity in older adults with cognitive impairments	Duplicate studies retrieved within or across databases
	Conference abstracts, protocol papers, case reports, commentaries/letters, dissertation/theses, books, and reviews (excluded due to insufficient methodological detail for charting)
	Articles without available full text
	Relevant literature that has not undergone peer review Articles not published in English

### 2.4 Data charting

Data were extracted using a pre-specified charting form aligned with the PCC framework and the review questions (Table 5). Extracted variables included study design, participant characteristics, telehealth modality and delivery model (e.g. facilitated/hybrid versus direct-to-home), setting, assessor discipline, assessment components (including any decision-specific capacity procedures), and reported outcomes (feasibility, reliability/validity, acceptability, barriers/opportunities). Given the limited and heterogeneous evidence base, findings were reported using a two-tier framework. Tier A included studies that explicitly assessed decision-making capacity for defined decisions/domains using telehealth (including comparisons with in-person assessment). Tier B included studies where telehealth cognitive/geriatric/psychiatric assessments informed capacity-adjacent recommendations but did not operationalise decision-making capacity as the primary outcome. In addition, telehealth delivery was coded as direct-to-home versus facilitated/hybrid models, as several studies involved onsite staff support for technology setup and, in some cases, components of assessments. A second reviewer (AB) verified extracted data for all included studies.

**Table 5:** Data charting template.

Item
Author
Country
Geographical area (e.g. urban, rural)
Study type (e.g. feasibility, validity)
Sample characteristics (e.g. age, sample size, ethnicity)
Modality of teleneuropsychology (e.g. telephone, videoconference)
Referral reason (e.g. dementia, capacity assessment)
Healthcare setting (e.g. outpatient clinic, hospital inpatient)
Instruments used (e.g. Montreal Cognitive Assessment, Test of Practical Judgment)
Clinical assessor (e.g. neuropsychologist, geriatrician etc.)
Main results
Opportunities
Barriers

## 3 Results

### 3.1 Literature search and selection

**Figure 1:**
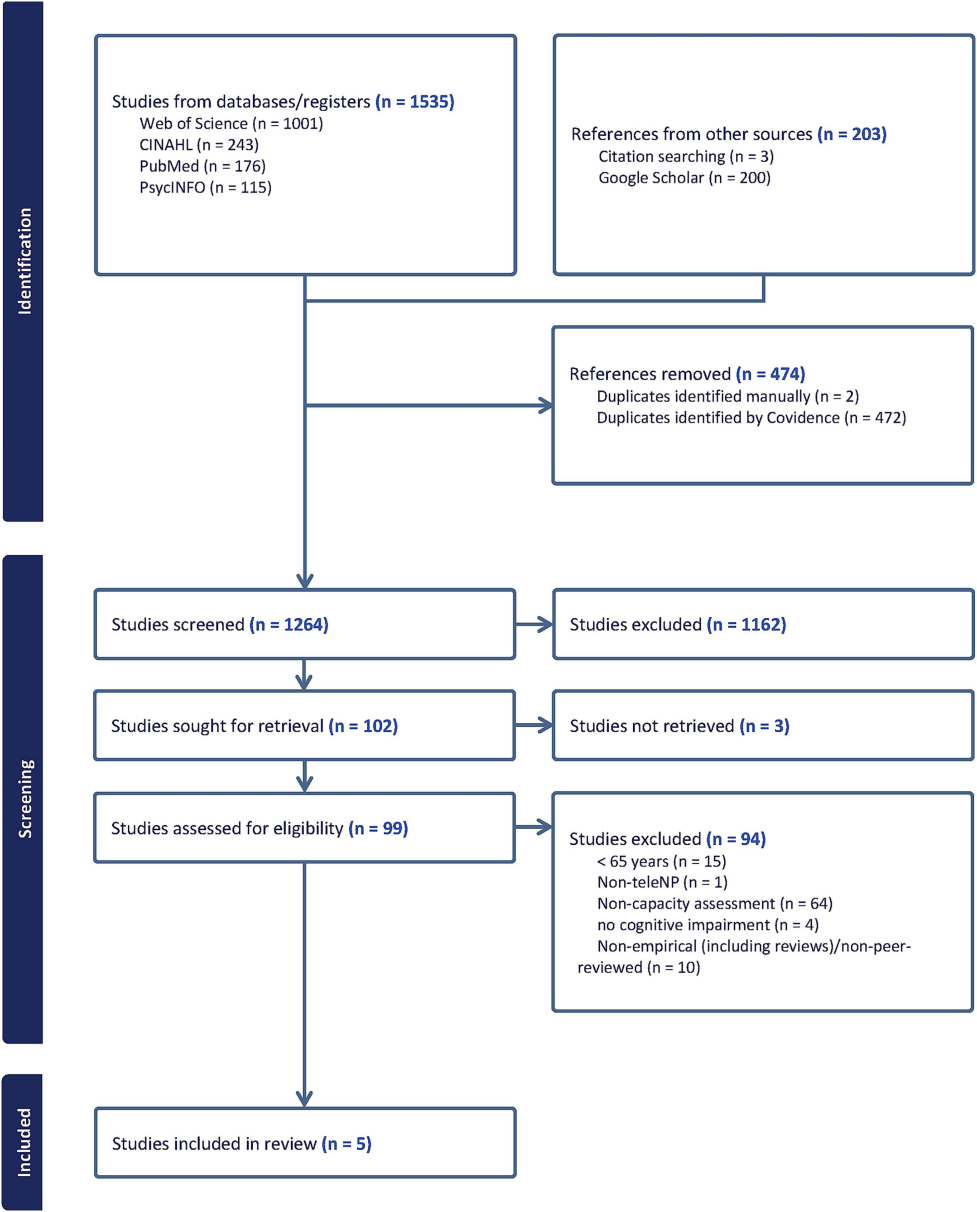
PRISMA flow diagram outlining the selection process followed to identify relevant studies for the review. Note: searches were updated on 05 Jan 2026. Titles/abstracts of newly indexed records were screened, and no additional eligible studies were identified. Therefore, the flow diagram reflects the 16 Apr 2024 fully processed screening workflow.

**Figure 2:**
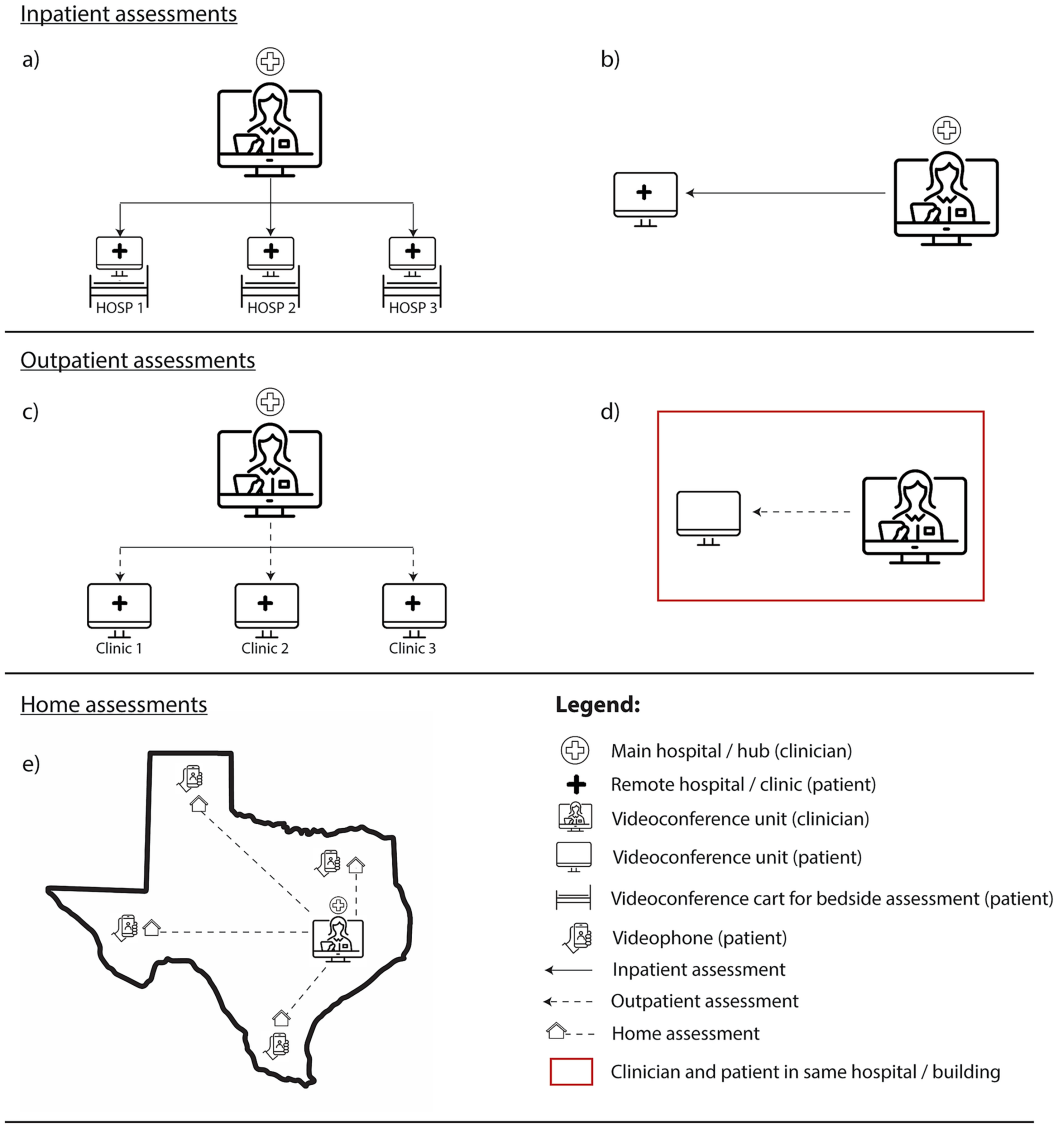
A schematic of the various health technology methods adopted across inpatient, outpatient, and home assessments.

An initial literature search across all five databases generated 1,738 records, consisting of 474 duplicates. Titles and abstracts of the remaining 1,264 records were screened against predetermined exclusion criteria and 102 full-text articles were subsequently retrieved. A total of 97 articles were excluded for the following reasons: unable to retrieve article (*n* = 3), participants <65 years (*n* = 15), non-teleneuropsychology methods (*n* = 1), non-capacity assessments (*n* = 64), studies involving participants without cognitive impairments (*n* = 4), and non-empirical/non-peer-reviewed studies (*n* = 10). A final shortlist of five articles was included in the review. Note, the database searches that were rerun on 05 Jan 2026 did not present additional eligible studies; therefore, the included study set remained *n* = 5. Figures 1 and 2 diagrammatically represents the search strategy flow of selecting studies from the chosen databases. Tables 6 and 7 summarise information extracted from the final papers using the data charting template.

**Table 6:** Characteristics of included studies and relevance to decision-making capacity (DMC) assessments in older adults with cognitive impairment.

Authors	Country	Setting/population	Telehealth modality and delivery model	Assessment focus	DMC tier and domain	Key outcomes (capacity/cognition)	Opportunities (O) and barriers (B)
Burnett et al. (2019)	United States	Adult Protective Services (APS) referrals; vulnerable adults/older adults	Videophone; direct-to-home with APS facilitation	Medical/mental health evaluation in context of abuse/neglect/exploitation; includes capacity	Tier A (explicit); guardianship/emergency removal contexts	Expanded assessment capacity (large increase in referrals); mental health assessments including capacity; substantial guardianship-related outcomes	O: Access to experts; timeliness; ability to view home environment; potential cost efficiency with distance B: Resourcing/scalability; infrastructure variability in remote areas; system bottlenecks beyond assessment
Gray et al. (2016)	Australia	Frail older inpatients in small rural hospitals	Videoconference; facility-facilitated (nurse supported)	Comprehensive geriatric assessment including cognition/ADLs; discharge/placement planning	Tier B (capacity-adjacent); accommodation/placement decisions	Feasible and sustained service model; high frailty and cognitive impairment captured	O: Feasibility/sustainability; cost effectiveness with long travel times; keeps complexity managed locally B: Low utilisation of inpatient geriatric assessments (in that model/population)
Harrell et al. (2014)	United States	Older veterans in remote clinics with suspected dementia	Videoconference; clinic-facilitated	Neuropsychological evaluation for diagnosis/differential and recommendations	Tier B (capacity-adjacent); driving, planning/POA, living support	High diagnostic clarification; uncovered unmet mental health needs; generated safety/planning recommendations	O: Improves access; high satisfaction; large travel savings B: Emergency protocols needed; technology barriers; reduced feasibility of some tests when conducted remotely; potential paranoia triggered
Johnston and Jones III (2001)	United States	Rural nursing facility residents	Videoconference; facility-facilitated (nurse supported)	Telepsychiatry consultations including cognitive screening; behavioural and psychiatric management	Tier A (explicit in at least one case); healthcare decision-making competency	Increased consultation volume; good engagement for most; orientation/hearing issues impacted some patients	O: Removes travel delays; increases clinician presence; nursing involvement supports implementation B: Low bandwidth issues affecting movement observations; staff training burden/turnover; hearing/audibility challenges
Martin-Khan et al. (2007)	Australia	Memory clinic patients	Videoconference; simulated remote (within hospital)	Dementia assessment plus structured capacity questions	Tier A (explicit); will-making; driving; living alone	Telehealth vs. face-to-face diagnostic agreement comparable; capacity agreement variable by domain	O: Supports feasibility/validity of videoconference for dementia assessments B: Equipment installation delays; some patients declined/participation barriers

**Table 7:** Telehealth assessment delivery models and implications for interpretation.

Authors	Assessor discipline	Onsite facilitator	Reported assessment components	Telehealth delivery model: interpretation notes
Burnett et al. (2019)	Geriatric experts	APS caseworker	History/medical review; observation-based physical exam; mental status/capacity assessment; functional and home environment evaluation; cognitive screening measures referenced	In-home facilitated model: The remote specialist assessment was supported by an onsite APS caseworker, who played a role in technology setup and contextual/functional observations. Findings reflect a service model with onsite support.
Gray et al. (2016)	Geriatricians	Local nurse/MDT	Structured geriatric assessment system (interRAI)	Facility facilitated model: Capacity-relevant information was largely generated onsite using a structured interRAI/geriatric assessment captured by local staff and with the remote specialist interpreting and advising. This study validates a workflow plus structured data capture model.
Harrell et al. (2014)	Neuropsychologist/fellow	Clinical staff implied (remote clinic site)	Standard neuropsychological battery (MoCA; WTAR; WAIS-IV Digit Span; Trails A/B; BNT; verbal fluency; RCFT; WMS Logical Memory; HVLT-R; BVMT-R; D-KEFS Proverbs; mood scales; plus add-ons)	Remote clinic facilitated model: Testing was delivered by videoconference but conducted at a remote clinic site with onsite staff support and tele-specific adaptations. Findings generalize to facilitated remote teleneuropsychology clinic.
Johnston and Jones III (2001)	Psychiatrist	RN/CNA/nursing staff	MMSE plus interview; nursing observations; physical exam by nursing staff; behavioural observations	Hybrid model: The psychiatric interview occurred remotely, while key components (e.g. physical exam and behavioural observations) were completed onsite by nursing staff. This study emphasises a combined remote and onsite assessment.
Martin-Khan et al. (2007)	Psychogeriatrician/Geriatrician	Nurse (pre-testing/workup)	Pre-interview cognitive tests (SMMSE, RUDAS, Clock, FAS, IQCODE); clinician interview via videoconference	Split workflow: Cognitive screening/tests were completed onsite (nurse-led pre-testing) with clinician interview/assessments via videoconference. Findings reflect partial tele-delivery layered onto an onsite assessment pathway.

Delivery labels (column 5) describe the degree to which assessment components were conducted remotely versus onsite. Facilitated models involve onsite support (e.g. technology setup, collateral information, or assistance with test materials) while the clinician assessment is delivered via telehealth. Hybrid models include substantive in-person clinical components (e.g. physical examination) in addition to remote consultation. Split workflow models involve an onsite pre-assessment workup (e.g. screening measures completed by staff) followed by a remote clinician interview/interpretation.

### 3.2 Country of research, geographical areas of service and healthcare setting

Three studies were conducted in the United States (Burnett et al., 2019; Harrell et al., 2014; Johnston and Jones III, 2001) and two in Australia (Gray et al., 2016; Martin-Khan et al., 2007). One provided teleneuropsychology services across urban and rural Texas (Burnett et al., 2019), while another operated in Brisbane, Australia (Martin-Khan et al., 2007). The remaining studies focused on rural regions in the United States (Harrell et al., 2014; Johnston and Jones III, 2001) and Australia (Gray et al., 2016). Two studies were inpatient-based (Johnston and Jones III, 2001; Gray et al., 2016), another two serviced outpatients (Harrell et al., 2014; Martin-Khan et al., 2007), and the remaining one uniquely assessed individuals at home (Burnett et al., 2019).

### 3.3 Assessment of decision-making capacity

Across the included studies, explicit operationalisation of decision-making capacity (Tier A) was uncommon and varied in specificity (Table 6). Three studies were classified as Tier A because they explicitly addressed capacity/competency or reported decision-specific capacity judgements in the assessment process (Burnett et al., 2019; Johnston and Jones III, 2001; Martin-Khan et al., 2007). However, the specificity of “capacity” procedures differed across these studies. One study most directly aligned with decision-specific framework by formally comparing videoconference versus in-person judgements across defined functional/decision domains (e.g. driving, living alone, making a will) (Martin-Khan et al., 2007). The remaining two Tier A studies embedded capacity evaluations within broader clinical service (i.e., protective services model) (Burnett et al., 2019) or service delivery contexts (i.e., facility-based telepsychiatry) (Johnston and Jones III, 2001), where capacity assessment was an explicit purpose or occurred in at least one case, but decision-specific methods were described with less granularity. As a result, although these studies referenced capacity/competency, they provided limited detail about how core capacity abilities (e.g. understanding, appreciation, reasoning, communication of choice) were elicited, supported, and documented for clinical-legal purposes.

Two studies were classified as Tier B because they did not operationalise capacity as a primary outcome (Harrell et al., 2014; Gray et al., 2016). Instead, they reported telehealth assessments that informed capacity-adjacent recommendations (e.g. safety, driving risk management, planning/guardianship considerations, or living support) without specifying decision-specific capacity assessment approaches, standards/thresholds, or the basis for any implicit capacity-related conclusions.

### 3.4 Assessment modality and clinical assessors

Videoconference was the predominant modality. Across studies, delivery was frequently facilitated, with onsite staff (e.g. nurses, caseworkers, or facility staff) supporting technology setup, providing collateral information, and in some cases assisting with assessment components.

### 3.5 Feasibility, reliability, and sustainability

Four studies examined the feasibility of teleneuropsychology methods (Burnett et al., 2019; Harrell et al., 2014; Johnston and Jones III, 2001; Gray et al., 2016), while one addressed reliability via comparison of videoconference versus face-to-face assessment (Martin-Khan et al., 2007), and one additionally reported service sustainability over time (Gray et al., 2016). Burnett et al. (2019) demonstrated rapid service implementation at scale, receiving 300 referrals within the first 8 months, with substantial use of virtual assessments to support case management. Gray et al. (2016) reported low overall utilisation relative to total admissions (1.2%), but noted the service disproportionately supported high-needs patients (accounting for 12% of bed use), many of whom had cognitive impairments and functional dependence. Harrell et al. (2014) reported clinical utility in a rural specialist model, including diagnostic clarification (87% diagnosis change) and identification of unmet mental health needs (77%), alongside reduced travel burden for patients. Johnston and Jones (Johnston and Jones III, 2001) observed increased consultation capacity over time (50% increase in consultation in 1 year, with many patients receiving multiple sessions). In the only study reporting reliability metrics, Martin-Khan et al. (2007) found substantial agreement between videoconference and face-to-face assessments (*k* = 0.63), comparable to agreement between two face-to-face assessments (*k* = 0.53). Sustainability was reported in the rural hospital geriatric model, with stable or increasing activity across sites after an initial implementation period (Gray et al., 2016).

### 3.6 Cost effectiveness and patient/clinician satisfaction

Only one study reported a formal cost analysis (Gray et al., 2016), finding telehealth consultations became more economical than in-person delivery once travel time exceeded a 76-min roundtrip and consultation volume reached a small threshold (four or more consultations). That model also described the workforce inputs required to deliver consultations, including nurse facilitation and specialist time. Other studies reported indirect efficiency gains, including reduced clinician travel and improved use of specialist time. Burnett et al. (2019) described reduced downtime associated with missed appointments when assessments were delivered virtually within an integrated service model, while Johnston and Jones (Johnston and Jones III, 2001) noted that eliminating travel effectively increased available consultation time within a fixed rural service contract. No studies reported validated patient or clinician satisfaction measures; however, acceptability signals were generally positive. Harrell et al. (2014) reported high engagement with assessment and feedback sessions and favourable informal feedback from patients and clinicians, and Johnston and Jones (Johnston and Jones III, 2001) described perceived benefits for patients and families from avoiding travel and enabling family involvement when appropriate.

### 3.7 Opportunities and barriers

The top two opportunities of telehealth identified across five studies were its feasibility in addressing referral questions and reduced travel time for patients and clinicians. Improved patient satisfaction, increased access to specialists, and cost-effectiveness were also noted. The primary barrier to adoption was poor technological infrastructure in rural areas. Harrell et al. (2014) highlighted challenges in administering certain neuropsychological tests, such as motor tasks and tests requiring frequent manipulation (e.g. Wisconsin Card Sorting Test, WAIS-IV Block Design), which are not yet feasible for telehealth. Concerns were raised about older patients’ discomfort with technology, potentially leading to increased anxiety and poor performance, thus affecting the construct validity of these tests.

## 4 Discussion

This scoping review mapped how telehealth has been used to support decision-making capacity assessments in older adults with cognitive impairments. The key finding is that direct evidence for telehealth-delivered capacity assessments remains limited: only three studies explicitly evaluated capacity judgements via telehealth for defined decisions/domains with just one of those comparing it to in-person outcomes (Burnett et al., 2019; Johnston and Jones III, 2001; Martin-Khan et al., 2007). The remaining two studies described telehealth cognitive/geriatric/psychiatric assessments that informed capacity-adjacent recommendations but did not operationalise decision-making capacity as the primary outcome (Harrell et al., 2014; Gray et al., 2016). While these Tier B studies provide important implementation signals (e.g. feasibility, staffing models, infrastructure requirements), they also underscore a major gap in decision-specific capacity procedures and reporting standards in telehealth contexts. The limited data around telehealth capacity assessments is in keeping with the Charles et al. (2024) online survey of Canadian clinicians involved in decision-making capacity assessments. Survey results indicated that virtual capacity assessments were relatively infrequent, despite more than half of the clinicians reporting interest in conducting such assessments. Barriers/facilitators clustered around patient/environment factors (e.g. patient communication difficulties), technology/technical support, and assessor capability to conduct capacity assessments virtually (e.g. observe behaviour/body language).

A second finding is that telehealth delivery was commonly facilitated or followed a hybrid model. Onsite staff support is a pragmatic feature of real-world telehealth for older adults (particularly in rural hospitals and residential facilities), but it complicates interpretation of what is being validated. Studies often evaluated a service model (e.g. remote specialist with onsite facilitation), rather than a fully remote, unassisted assessment.

The review also highlights the need to strengthen conceptual clarity around how neuropsychological evidence contributes to decision-making capacity assessments. Notably, most included studies did not feature neuropsychologists as the primary assessors, and capacity-related judgements were often embedded within telegeriatric, telepsychiatric, or other service models. This pattern suggests that the current evidence base reflects how capacity-related decisions are supported in routine geriatric care pathways, rather than tele-neuropsychology-led capacity assessments specifically. It also implies that future telehealth-based capacity models are likely to be most effective when designed as multidisciplinary services, where neuropsychology input is available when indicated but capacity assessment and decision support may be delivered by other appropriately trained clinicians within clear governance and reporting standards. As such, cognitive tests should not be framed as determinative thresholds for capacity. Instead, telehealth protocols should specify decision-specific interview procedures aligned to core decision-making capacity elements (e.g. understanding, appreciation, reasoning, communication; emotion and personal values), indicate how cognitive findings are integrated as supportive evidence, and describe how collateral information and functional observations are incorporated. For future research, this highlights the need for clearer reporting of which professional discipline conducts each assessment component, how cognitive evidence is generated and interpreted, and whether neuropsychology-led tele-assessments add incremental value (e.g. improved decision-specific reasoning documentation, identification of cognitive mechanisms underlying decisional difficulty, and greater consistency and transparency of tribunal-ready reporting) relative to broader telehealth consultation models. Evaluation of the need for teleneuropsychology specific normative data may also be necessary.

Finally, the limited recency of included studies suggests that telehealth practice may be advancing faster than the published literature on decision-making specific applications. Priorities for future work include (i) operationalising tele decision-making specific assessment procedures for defined decision domains, (ii) evaluating feasibility and acceptability for older adults with cognitive impairments (including sensory/functional impairments and communication needs), (iii) examining reliability/validity of tele-delivered decision-making specific judgements (including borderline cases), and (iv) developing minimum reporting standards for telehealth capacity evaluations that clearly describe delivery model, facilitation, safeguards, and documentation processes.

## 5 Limitations

This review has its limitations. First, only a subset of records was independently screened by second reviewers. To improve transparency, we note that agreement between the reviewer and verifiers was 98.5% for title/abstract screening and 100% for full-text decisions, and extracted data were verified. Nevertheless, because duplicate screening was not undertaken for all title/abstract records, some risk of missed studies remains. Second, restricting inclusion to peer-reviewed English-language publications may have excluded relevant work in other languages and recent evidence reported in conference abstracts, theses, or service evaluations. This is particularly important in a rapidly evolving field such as telehealth, where emerging models may first be described in conference abstracts, theses, service evaluations, implementation reports, or other non-indexed sources, including those arising in technologically advanced non-English-speaking and culturally and linguistically diverse settings. Given the small evidence base identified, future scoping work may benefit from explicitly searching and charting grey literature to improve sensitivity to emerging models of telehealth capacity assessments. Third, heterogeneity in telehealth delivery models and variability in reporting limited the ability to draw strong conclusions about the validity of telehealth-delivered decision-making capacity assessment procedures.

## 6 Conclusion

There is limited published evidence on telehealth decision-making capacity assessments in older adults with cognitive impairments. While teleneuropsychology and related telehealth assessment models show promise for improving access and timeliness – particularly in facilitated/hybrid service models – most published work does not operationalise decision-making capacity as a primary outcome or describe decision-specific capacity procedures and documentation standards. Future research should prioritise clear operationalisation and reporting of decision-specific telehealth decision-making capacity protocols, delivery models (including facilitation), and outcomes addressing feasibility, reliability/validity, and stakeholder acceptability.

## Data Availability

The original contributions presented in the study are included in the article/Supplementary material, further inquiries can be directed to the corresponding author.
